# Understanding CT Perfusion in Acute Ischemic Stroke: How Algorithms Shape Perfusion Maps

**DOI:** 10.3390/diagnostics16121831

**Published:** 2026-06-12

**Authors:** Nicola Morelli, Marco Spallazzi, Marina Biondi, Eugenia Rota, Davide Colombi

**Affiliations:** 1 Neuroradiology Unit, Guglielmo da Saliceto Hospital, 29121 Piacenza, Italy; m.biondi@ausl.pc.it; 2 Neurology Unit, University of Parma, 43121 Parma, Italy; mspallazzi@gmail.com; 3 Neurology Unit, San Giacomo Hospital, 15067 Novi Ligure, Italy; eugenia.rota.md@gmail.com; 4 Department of Diagnostic Imaging, Centro Diagnostico Rocca, 29121 Piacenza, Italy; colombidavide@gmail.com

**Keywords:** CT perfusion, acute ischemic stroke, deconvolution, perfusion imaging, Bayesian algorithm, Tmax, ischemic core, stroke imaging

## Abstract

CT perfusion (CTP) is widely used in acute ischemic stroke imaging, particularly for treatment selection beyond conventional time windows. However, automated perfusion maps are not direct measurements of irreversible tissue injury, but estimates shaped by deconvolution strategy, temporal correction, dispersion handling, and software-specific thresholds. This review provides a clinically oriented explanation of how CTP algorithms influence the estimation of ischemic core and hypoperfused tissue. Particular attention is given to singular value decomposition (SVD) methods, Bayesian approaches, and timing parameters, including time to maximum (Tmax), Delay, time to peak (TTP), and mean transit time (MTT). Differences in residue function estimation and threshold definition may generate variable outputs across software platforms, even from the same source dataset. Perfusion thresholds should therefore not be treated as universally interchangeable. CTP findings should be integrated with clinical status, non-contrast CT, CT angiography (CTA), collateral status, occlusion site, and imaging-to-treatment context, serving as decision-support tools rather than isolated measures of tissue viability.

## 1. Introduction

CT perfusion (CTP) has evolved from a research tool into a clinically relevant component of acute ischemic stroke imaging. In current stroke workflows, CTP may contribute to treatment selection for mechanical thrombectomy in extended time windows and to intravenous thrombolysis in selected patients beyond conventional time limits [1,2,3]. As a result, automated perfusion maps are increasingly incorporated into rapid clinical decision-making.

In daily practice, clinicians are often required to estimate how much brain tissue is already irreversibly injured and how much remains potentially salvageable. In general, automated CTP post-processing platforms provides rapid estimates of ischemic core and hypoperfused tissue, usually displayed as color-coded maps. However, these outputs are not direct measurements of permanent tissue injury. Rather, they are generated through mathematical models, deconvolution procedures, temporal correction strategies, and software-specific thresholds. Consequently, the same source dataset may produce different estimates of core and penumbra when processed with different platforms or algorithms [4,5,6].

This algorithmic dependency has practical clinical implications. Perfusion maps may be influenced by assumptions regarding arterial input function selection, bolus delay, bolus dispersion, residue function estimation, regularization strategies, and threshold definition. These factors may affect the apparent extent of ischemic core and hypoperfused tissue, particularly in patients with proximal arterial occlusion, collateral-dependent flow, or delayed contrast arrival.

Although CTP is widely used in acute stroke imaging, the algorithmic foundations of perfusion maps are often addressed only briefly in clinically oriented literature and are seldom presented as a practical interpretive framework for radiologists, neuroradiologists, and stroke physicians. The aim of this review is to provide a clinically accessible explanation of how algorithms shape CTP maps, with particular attention to deconvolution models, delay and dispersion effects, Bayesian approaches, and the interpretation of time-related perfusion metrics in acute ischemic stroke.

Rather than providing an exhaustive vendor-by-vendor comparison of perfusion thresholds, this review explains why such thresholds are algorithm-dependent and should be interpreted within their specific processing context.

## 2. Literature Search Strategy

A targeted literature search was performed to identify relevant methodological, technical, and clinical studies on CTP in acute ischemic stroke. PubMed/MEDLINE, Scopus, Web of Science, and Google Scholar were searched. Search keywords included “CT perfusion”, “acute ischemic stroke”, “deconvolution”, “singular value decomposition”, “Bayesian”, “Tmax”, “Delay”, “ischemic core”, “ghost core”, “software thresholds”, and “stroke imaging”, used in different combinations. Priority was given to methodological studies, comparative software studies, validation studies, clinical guidelines, and recent publications, while older foundational articles were retained when essential for explaining the mathematical and physiological basis of CTP.

## 3. From Densitometry to Mathematical Models

**Figure 1:**
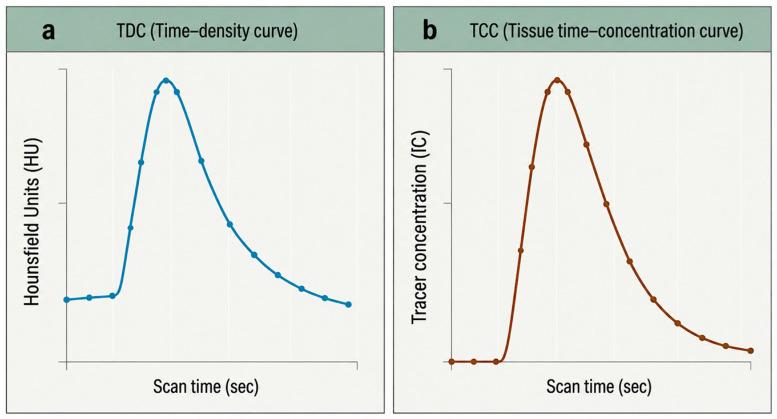
*Relationship between attenuation-based time–density curves (TDCs) and tissue time–concentration curves (TCCs) in CT perfusion*. (**a**) Attenuation-based TDC expressed in Hounsfield units. (**b**) Baseline-corrected TCC expressing tracer concentration over time. Although TDCs and TCCs exhibit similar morphology, they differ in baseline definition. In dynamic CT perfusion acquisitions, image sampling begins after contrast injection and includes initial *baseline time points* acquired before visible contrast arrival. In attenuation-based TDCs, signal values therefore do not start from zero because baseline attenuation reflects the intrinsic density of the tissue before contrast arrival. In contrast, tissue concentration curves represent contrast concentration over time, which is assumed to be zero before contrast arrival. Assuming a *linear relationship* between attenuation change and contrast concentration, C(t) can be derived from baseline-corrected attenuation values according to C(t) = k × [S(t) − S(b)], where S(t) is the measured attenuation signal, S(b) is the baseline attenuation, and k is a proportionality constant dependent on acquisition parameters, contrast delivery, vascular architecture, and patient-specific factors.

CTP is based on a simple principle: the brain is scanned repeatedly during the passage of an iodinated contrast bolus through the cerebral vasculature, and attenuation changes are measured over time. These measurements generate *time–density curves* (TDCs), expressed in Hounsfield units (HU), which describe signal variation in *arteries*, *tissue*, and *veins*. Assuming a linear relationship between attenuation and contrast concentration, TDCs can be converted into time–concentration curves, hereafter referred to as *tissue concentration curves* (TCCs) [7,8]. Although TDCs and TCCs are often used interchangeably because of their similar morphology, they are conceptually distinct, and subsequent perfusion analysis is based on tissue contrast concentration over time (Figure 1).

The *arterial input function* (AIF) is obtained from arterial voxels, usually within large intracranial arteries, whereas *tissue concentration curves* are sampled within the brain parenchyma. Arterial measurements may be affected by partial-volume effects, particularly in small vessels or when spatial resolution is limited [9,10]. Modern software increasingly uses automated vessel detection or multi-candidate arterial sampling to identify a robust input curve. The *venous output function* (VOF), typically obtained from a large venous structure such as the superior sagittal sinus, is less susceptible to these artifacts and may be used as a reference for normalizing quantitative perfusion parameters, thereby reducing amplitude errors related to partial-volume averaging.

The *tissue concentration curve* resembles the arterial input but also reflects bolus dispersion through the vascular tree and microvascular dynamics. It should therefore be regarded as a temporally filtered version of the arterial signal rather than a direct replica. To translate these curves into quantitative perfusion parameters, mathematical modeling is required. *Convolution* and *deconvolution* form the computational basis of CTP analysis and underlie the perfusion maps ultimately used in clinical decision-making.

## 4. Convolution and Deconvolution: Understanding the Mathematical Basis of Perfusion

In mathematics and signal processing, *convolution* is an operation in which two functions are combined to generate a third. In CTP, the arterial input function, AIF(t), and the residue function, R(t), combine to produce the tissue concentration curve, C(t), observed over time [11,12].

The arterial input function describes contrast delivery to the brain, whereas the residue function reflects the *microvascular response of the tissue*. More intuitively, R(t) can be understood as the modeled fraction of contrast remaining within a tissue voxel over time after an idealized instantaneous bolus; it ideally starts at 1 and decays toward 0 as contrast leaves the tissue, and therefore represents the hemodynamic quantity of greatest interest. Conceptually, *convolution* (⊗) can be viewed as the process by which the arterial input is transformed by the tissue microvasculature into the measured tissue curve. In CTP, this relationship can be expressed as

C(t) = AIF(t) ⊗ R(t)

Whereas C(t) and AIF(t) are directly sampled from dynamic CT data, R(t) is not measured directly but inferred mathematically. Recovering R(t) from the measured tissue concentration curve and the known AIF requires *deconvolution*, that is, the solution of an *inverse problem*:

R(t) = deconv [C(t), AIF(t)]

This operation is not a simple division, but an estimation of a hidden physiological process from a combined and noisy signal. In clinical CT perfusion, this inversion is commonly performed using *singular value decomposition* (SVD), a classical linear algebra technique that decomposes a matrix into simpler components. Both the *arterial input function* and the *tissue concentration curves* can be represented in matrix form, and SVD allows estimation of the residue function while reducing the influence of noise and numerical instability [13,14,15]. From a practical perspective, SVD converts temporally blurred and noisy enhancement curves into estimated perfusion parameters, such as cerebral blood flow, cerebral blood volume, mean transit time, and Tmax.

**Figure 2:**
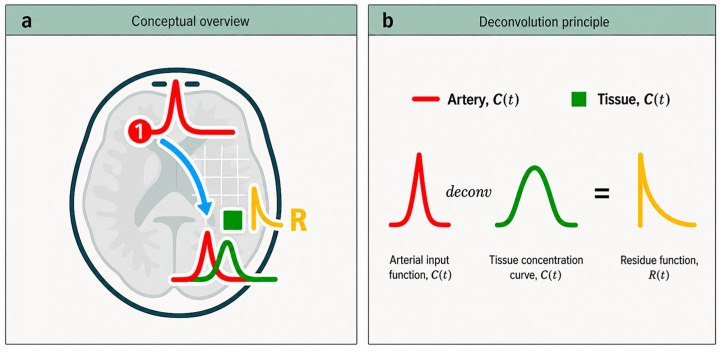
*Conceptual diagram of perfusion deconvolution and residue function estimation*. The notation is intentionally simplified for educational purposes. (**a**) Conceptual overview showing the arterial input function (AIF) and the tissue concentration curve, C(t). The arterial curve displayed near the tissue region is identical to the AIF and is rescaled for visual comparison. The blue arrow schematically indicates the path from the arterial input measurement site to the selected tissue voxel, rather than a directly measured anatomical distance. (**b**) Deconvolution principle: the *arterial input function* and *tissue concentration curve* are the primary measurable signals; deconvolution estimates the *residue function* R(t), from which major perfusion parameters are derived.

In summary, convolution explains how AIF(t) and the tissue residue response combine to generate the observed C(t). Deconvolution describes how the hidden tissue response is estimated from AIF(t) and C(t), and SVD provides one of the main practical tools for this reconstruction. The conceptual relationship among AIF(t), C(t), and R(t) is illustrated in Figure 2.

## 5. Delay Sensitivity and Dispersion

One of the main challenges in CT perfusion is the temporal mismatch between contrast arrival in a proximal artery, where the arterial input function is sampled, and contrast arrival within distal brain tissue. This *delay* may result from proximal arterial obstruction, collateral pathways, or differences in vascular path length [16].

If deconvolution is performed without accounting for this temporal shift, delayed contrast arrival may be misinterpreted as reduced blood flow. As a consequence, part of the delay may be incorporated into perfusion estimates, leading to systematic misestimation of tissue perfusion and distortion of derived perfusion maps [17].

On this basis, deconvolution algorithms can be broadly divided into two categories.

*Delay-sensitive algorithms*. These methods do not adequately compensate for temporal mismatch between the arterial input function and tissue curves and therefore remain vulnerable to delay-related bias.

*Delay-corrected algorithms*. By contrast, these approaches attempt to address temporal misalignment through realignment or correction strategies that reduce delay-induced distortion of perfusion estimates, although residual bias may persist in severe or complex hemodynamic conditions [18,19].

A related but distinct phenomenon is bolus *dispersion*. While *delay* refers to a temporal shift in contrast arrival, *dispersion* refers to progressive broadening and reshaping of the contrast bolus during vascular and microvascular transit. In dispersed-flow conditions, the tissue concentration curve is not only delayed but also wider and lower in peak amplitude than the arterial input. This can affect estimates of flow, timing, and tissue viability, particularly in collateral-dependent or watershed territories.

The distinction between *delay* and *dispersion* is clinically relevant because different algorithms handle these phenomena differently. Some approaches mainly reduce delay sensitivity, whereas others attempt to model both delay and dispersion. Understanding this distinction helps explain why perfusion maps may exaggerate or underestimate ischemic injury depending on the processing method used, especially in patients with proximal arterial occlusion or complex collateral circulation.

## 6. Direct and Deconvolution-Derived Parameters

*Deconvolution-derived parameters*. The residue function, R(t), obtained through deconvolution, is generally regarded as the closest mathematical representation of *tissue microvascular behavior*. Several key perfusion parameters are derived from its temporal profile, because the height, shape, and area of the deconvolved response encode different aspects of tissue perfusion, including flow amplitude and contrast transit through the microvascular compartment. Cerebral blood flow (CBF), expressed in mL/100 g/min, reflects the amount of blood passing through a unit volume of brain tissue per unit time and is derived from the maximum height of the deconvolved residue response. Mean transit time (MTT), measured in seconds, represents the average time that blood spends within the capillary network and is related to the area under R(t). According to the central volume theorem, MTT is proportional to the ratio between cerebral blood volume (CBV) and CBF. Tmax represents the time to the maximum of R(t) and provides a model-derived timing metric of the tissue residue response [20,21,22,23,24].

**Figure 3:**
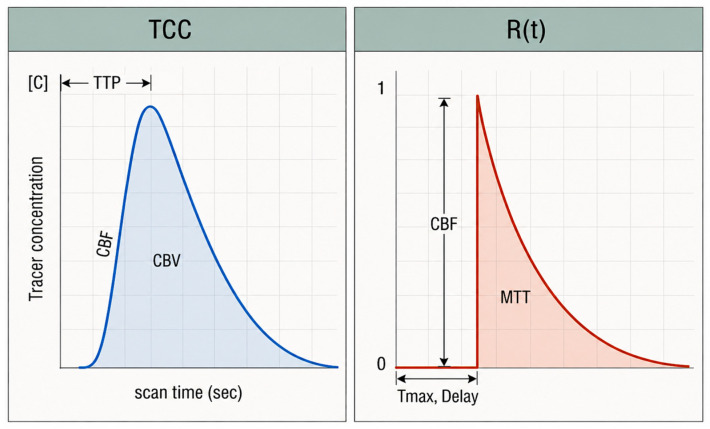
*Derivation of CT perfusion parameters from the tissue concentration curve (TCC) and the deconvolved residue function, R(t)*. In the (**left panel**), CBV and classic TTP are derived directly from the tissue concentration curve, C(t). The CBF label on the upslope of C(t) denotes a slope-based approximation. In the (**right panel**), deconvolution yields the *residue function*, R(t), from which CBF, MTT, and Tmax are derived. Because the theoretical residue function is normalized to an initial value of 1, the deconvolved *flow-scaled response*, CBF × R(t), has a maximum height corresponding to CBF. Depending on the algorithm, Delay may be estimated from, or incorporated within, the deconvolution model. Tmax and Delay are shown together only for illustrative purposes, reflecting an *idealized residue function* with an instantaneous vertical rise from 0 to 1, in which the onset and maximum occur at the same time. However, in *physiological perfusion* data, R(t) does not rise vertically but may show a progressive upslope over time. *Delay* more closely reflects *arterial–tissue arrival delay*, corresponding schematically to the onset of R(t), whereas *Tmax* reflects the time required for R(t) to reach its *maximum* and is influenced by its shape, including MTT-related broadening. Therefore, Tmax may occur after Delay and should not be considered directly interchangeable with it.

*Directly derived parameters*. By contrast, some perfusion parameters are derived directly from the tissue concentration curve, C(t), without deconvolution. CBV, expressed in mL/100 g, is derived from the area under the tissue concentration curve after vascular input normalization and reflects the total volume of blood contained within a given tissue voxel during contrast passage. Time to peak (TTP) represents the time point at which C(t) reaches its maximum value. Because no residue function is estimated, these parameters retain a more immediate relationship with the measured contrast signal. Their distinction from deconvolution-derived parameters, including their different mathematical origin, algorithmic dependence, sensitivity to temporal features of the measured curve, and susceptibility to acquisition-related factors, is summarized in Table 1 and illustrated in Figure 3.

## 7. Deconvolution Algorithms: One Dataset, Many Possible Outputs

In clinical practice, deconvolution is not performed by direct algebraic solution but by numerical algorithms that approximate the *inverse problem* through matrix operations and regularization strategies. In this context, *regularization* refers to mathematical constraints or stabilization procedures introduced to limit noise amplification and avoid unstable or non-physiological estimates of R(t). This step is necessary because small fluctuations in the sampled arterial input function or tissue concentration curves may be amplified during inversion, producing irregular residue functions and less reliable parameter maps.

Regularization therefore represents a balance between preserving physiologically meaningful signal and suppressing noise-related instability. Although it improves numerical robustness, different choices may subtly modify the recovered residue function and the parameters derived from it [25].

The practical implication is straightforward: when the same dataset is processed with different algorithms, the resulting perfusion maps may not be identical. Each method relies on specific assumptions, levels of noise tolerance, and strategies for handling delay and dispersion; therefore, parameters such as CBF, MTT, Tmax, and Delay may vary even when derived from the same source data [26]. This variability becomes clinically relevant when these parameters are translated into estimates of ischemic core, hypoperfused tissue, or mismatch volume, particularly in patients close to treatment eligibility thresholds. In such cases, small differences in map generation may influence the apparent balance between irreversibly injured and potentially salvageable tissue.

**Figure 4:**
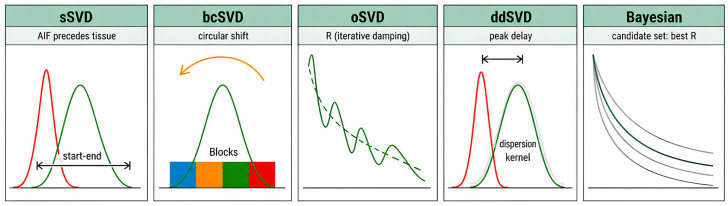
*Models of perfusion deconvolution*. For illustrative purposes, diagrams show either the time–concentration curve C(t) or the residue function R(t), depending on which best conveys the principle of each method. In all deconvolution-based approaches, the goal is to estimate R(t). **sSVD**: *Linear deconvolution* with fixed temporal boundaries; assumes that the arterial input precedes the tissue curve, making it simple and fast but *delay-sensitive*. **bcSVD**: *Block-circulant formulation* in which the convolution matrix imposes a circular boundary condition, mathematically linking the end of the time curve to its beginning. This improves numerical stability and reduces delay sensitivity. The colored blocks are schematic only and refer to the block structure of the convolution matrix, not to a temporal fragmentation of the tissue curve into separate time segments. **oSVD**: Extension of bcSVD with *oscillation-index regularization*, meaning that non-physiological oscillations in R(t) are suppressed to obtain smoother and more stable residue functions, at the cost of higher computational demand and possible over-smoothing. **ddSVD**: Explicit correction of delay and dispersion; *delay* is addressed by temporally aligning the arterial input with the tissue curve, while dispersion is modeled using a vascular *dispersion kernel* that describes the temporal spreading and smoothing of the bolus between the arterial input and the tissue response. The gray band around the tissue curve schematically represents this dispersion effect, accounting for the tissue curve being broader, smoother, and lower in peak amplitude than the arterial input. This may yield a more physiologic estimate of R(t), at the cost of greater model complexity. **Bayesian**: *Probabilistic inference* under physiological constraints, yielding the most plausible estimate of R(t) given the observed data, with robustness to noise and truncation, but with greater reliance on prior assumptions and parameter settings.

The main characteristics of the principal deconvolution methods are summarized in Table 2, and a simplified graphical overview is provided in Figure 4.

### 7.1. Standard Singular Value Decomposition

Standard singular value decomposition (sSVD) estimates the residue function R(t) by modeling the tissue concentration curve as the convolution of the arterial input function with R(t). In its standard implementation, deconvolution is formulated as a linear temporal problem, in which the acquired time curve has a defined beginning and end within the sampling window. Singular values representing dominant system components are retained, whereas values below a fixed threshold are discarded as noise [7]. This threshold acts as a regularization parameter: a lower threshold preserves more low-amplitude signal but may amplify noise, whereas a higher threshold suppresses noise more strongly but may remove physiologically relevant information, particularly in low-flow or delayed regions.

Because the temporal window is treated linearly, sSVD is vulnerable to boundary effects and to temporal mismatch between the sampled arterial input and the tissue curve. The method assumes that tissue enhancement cannot precede, and should closely follow, the sampled arterial input function. When this assumption is violated, as may occur in collateral-dependent circulation or delayed bolus arrival, temporal mismatch may be incorporated into the deconvolution process, introducing bias into the resulting perfusion estimates. Its main advantages are simplicity and computational efficiency, but its delay sensitivity limits reliability in complex hemodynamic settings.

### 7.2. Block-Circulant Singular Value Decomposition

Block-circulant singular value decomposition (bcSVD) was developed to reduce the delay sensitivity and boundary-related instability of standard SVD. Instead of treating the acquired time curve as a purely linear sequence with fixed temporal boundaries, bcSVD adopts a *circular formulation* of the convolution matrix. In this framework, the end of the signal is mathematically linked to its beginning. This does not represent a physiological assumption about contrast recirculation, but rather a mathematical construct designed to reduce boundary-related artifacts and improve numerical stability in the presence of delayed bolus arrival [12].

This *circular assumption* is implemented through a *block-circulant matrix* structure, in which the convolution operator is represented in coupled sub-blocks rather than as a purely linear temporal system. Although non-physiological, it makes the method substantially less sensitive to bolus delay. bcSVD therefore reduces the risk of systematic bias in delayed conditions while preserving computational efficiency. Its limitations derive from the same circular construct, which may introduce artifacts or oversimplify the underlying vascular behavior. In the literature, the same circular reformulation is also commonly described more broadly as circulant SVD (cSVD), particularly when the block structure is not explicitly emphasized.

### 7.3. Oscillation-Index Regularized SVD

Oscillation-index regularized SVD (oSVD) builds on the block-circulant approach by adding oscillation-index regularization. After an initial *voxel-wise* deconvolution, non-physiological oscillations in the estimated R(t), often referred to as *ringing artifacts*, are quantified, and the singular value threshold is *iteratively* adjusted until they fall within an acceptable range [4]. This generally improves map stability, particularly in noisy or motion-degraded datasets.

Because it is based on a block-circulant formulation, oSVD is also less sensitive to bolus arrival delay than standard SVD. However, it does not explicitly correct for bolus dispersion, so flow and temporal parameters may still be affected in dispersed-flow conditions. Greater computational demand and possible over-smoothing are additional limitations.

### 7.4. Delay- and Dispersion-Corrected SVD

Delay- and dispersion-corrected SVD (ddSVD) attempts to address two major sources of error: bolus delay and bolus dispersion. *Delay* is typically corrected by *cross-correlation*, shifting the arterial input function along the time axis until it is optimally aligned with the tissue curve [27]. *Dispersion* is modeled by incorporating a *vascular dispersion kernel*, or transport function, that describes how the contrast bolus progressively broadens and decreases in peak amplitude during vascular and microvascular transit. As a result, the tissue concentration curve becomes delayed, flatter, and more prolonged than the arterial input curve.

By correcting for both effects, ddSVD can provide a residue function that more closely reflects voxel-level hemodynamics, particularly in collateral-dependent or watershed territories where simpler methods may lead to less reliable estimates of infarct core. These advantages come at the cost of greater computational complexity, stronger model dependence, and less intuitive interpretation for non-specialists.

### 7.5. Bayesian Deconvolution

Bayesian deconvolution adopts a different framework, treating perfusion analysis as a *probabilistic inference* problem rather than a direct algebraic inversion. Using Bayes’ theorem, it estimates the *most plausible* residue function given the observed data and prior physiological constraints [28,29]. Instead of relying only on thresholded matrix inversion, Bayesian models incorporate biologically plausible assumptions, such as a residue function that starts at unity, remains non-negative, and decays monotonically.

Candidate solutions are evaluated probabilistically, and the solution with the highest posterior probability is selected. This approach is generally robust in noisy, truncated, or motion-affected datasets, where conventional SVD-based methods may be less stable. Its main limitations are potentially greater computational complexity, dependence on prior assumptions, and the need for a probabilistic rather than deterministic interpretative approach.

## 8. Time-Based Perfusion Metrics: TTP, MTT, Delay, and Tmax

Several time-related parameters are used in CT perfusion imaging, each reflecting different aspects of contrast kinetics and relying on distinct mathematical assumptions [20,21,22,23,24]. Although often interpreted together in clinical practice, they are not interchangeable, because they may derive from different curves, models, and correction strategies. This section focuses on the parameters most encountered in clinical stroke CTP interpretation: TTP, MTT, Delay, and Tmax.

### 8.1. Time to Peak

Classic time to peak (TTP) is a qualitative parameter derived directly from the tissue concentration curve, C(t). It represents the time point at which tissue enhancement reaches its maximum. Because it does not account for arterial–tissue timing differences, TTP is highly sensitive to bolus delay and may be vulnerable to error when contrast arrival is late, such as in proximal stenosis or collateral-dependent flow [23,24].

### 8.2. Model-Based TTP

In some model-based implementations, TTP may be estimated from a regularized, noise-reduced tissue curve rather than from the raw tissue concentration curve. This may improve robustness to noise and simple delay effects, although the exact implementation may vary across software platforms [30]. Therefore, model-based TTP may provide a cleaner, less noise-sensitive estimate than classic TTP.

### 8.3. Mean Transit Time

Mean transit time (MTT) represents the average time that blood spends within the tissue microvascular compartment. It therefore describes the duration of tissue transit rather than the onset of contrast arrival or the timing of maximal enhancement [20,21,22].

MTT can be estimated in two conceptually different ways. In *non-deconvolution approaches*, it is usually obtained indirectly from the central volume theorem, as the ratio between CBV and CBF. In this setting, CBV is derived from the tissue concentration curve, whereas CBF may rely on slope-based or other parametric methods; consequently, MTT remains an indirect parameter, dependent on how both CBV and especially CBF are estimated. With *deconvolution*, by contrast, MTT is derived from the residue function, R(t), and corresponds to the area under this function. This approach is generally considered more robust because it relies on the tissue residue response rather than on a slope-based approximation [20,21,22].

A prolonged MTT widens the tissue response and may contribute to a later peak of the residue function. For this reason, MTT is an important determinant of time-based metrics that depend on the shape of R(t), particularly Tmax [20,21,22,23,24].

### 8.4. Delay

Delay refers to the temporal shift between the *arterial input function* and the *tissue concentration–time curve*, C(t), reflecting *arterial–tissue arrival delay*. It is intended to capture the arrival-time difference between the arterial reference curve and the tissue curve, rather than the time at which the deconvolved residue function reaches its maximum. In this sense, Delay is closer to an arrival-time metric [16,17,18,19]. In some implementations, this parameter is also referred to as Delay Time (DT).

Delay is particularly relevant when it is explicitly incorporated into the deconvolution model, as in ddSVD and Bayesian approaches. In these settings, Delay is not simply a map calculated after R(t) has been estimated, nor a secondary correction applied to already-derived perfusion parameters. Rather, it is part of the model used to temporally align the arterial input function with the tissue response, thereby allowing a more appropriate estimation of R(t) and of the parameters derived from it [18,19,28,29].

### 8.5. Tmax

Tmax represents the time to maximum of R(t), estimated after deconvolution. It is therefore a model-derived metric, not a direct physiological measurement. It should not be confused with the peak of the raw tissue concentration curve or with the simple onset of contrast arrival [31]. Tmax should be regarded as a composite timing metric rather than a pure measure of arterial–tissue arrival delay. Its value depends not only on bolus arrival delay but also on the shape of R(t). Delay shifts the tissue response in time, dispersion broadens and smooths it, and prolonged MTT widens the residue response. Together, these factors may move the maximum of R(t) further to the right.

When calculated with standard SVD, Tmax may be particularly vulnerable to delay and dispersion effects. Algorithms designed to reduce delay-related bias may provide more robust estimates [12,31].

In practical terms, Delay and Tmax describe related but physiologically and mathematically distinct temporal aspects of tissue perfusion. They would coincide only in the idealized case of an instantaneous vertical rise in R(t); in *physiological* perfusion data, Tmax is generally expected to occur after the onset captured by Delay because it is influenced by the shape of R(t), including dispersion and MTT-related broadening. Therefore, Tmax and Delay should not be considered interchangeable, and their thresholds remain processing-dependent [31]. In ddSVD and Bayesian frameworks, Delay may serve as the primary timing parameter, whereas Tmax, when reported, may be derived as a model-based estimate rather than identified as the direct maximum of a numerically reconstructed R(t).

## 9. Timing, Thresholds, and Software Variability

Once perfusion parameters such as CBF, CBV, MTT, Tmax, Delay, and TTP have been defined, the next step is to understand how automated postprocessing translates them into operational thresholds for ischemic core and hypoperfused tissue. In clinical practice, ischemic core is commonly estimated using relative cerebral blood flow (rCBF) thresholds, whereas hypoperfused tissue is more often defined by Tmax or delay criteria [23,24,31]. One of the most widely used examples is RAPID, which applies rCBF < 30% for ischemic core and Tmax > 6 s for hypoperfused tissue. With this approach, penumbra is operationally estimated as the mismatch between the hypoperfused volume defined by Tmax and the core defined by rCBF.

Other applications may apply different operational criteria. For example, MIStar, based on ddSVD processing, may use DT > 3 s for hypoperfused tissue, a threshold reported to show good operational concordance with Tmax > 6 s. Olea Bayesian implementations have also been reported to use relative CBF < 25% for ischemic core and differential TTP > 5 s as a marker of delayed tissue transit. Vitrea has been reported to include different processing options, including SVD+ and Bayesian estimation. These examples illustrate that thresholds should be interpreted within the processing environment in which they were defined, rather than as universal physiological values. This distinction is particularly relevant when patients are close to core or mismatch eligibility limits.

**Figure 5:**
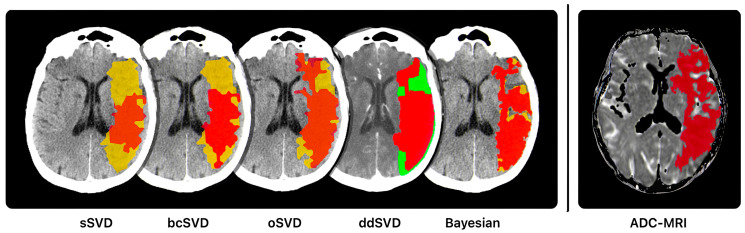
*Variability of perfusion maps in an illustrative case of middle cerebral artery occlusion imaged in an extended treatment window*. A single source dynamic perfusion dataset was processed with different deconvolution algorithms, arranged from left to right according to progressively more comprehensive correction for delay and dispersion: sSVD, bcSVD, oSVD, ddSVD, and Bayesian. Color overlays indicate ischemic core in red and penumbra in yellow or green; lesion volumes vary across algorithms and appear differently aligned with the diffusion-defined lesion, highlighting the model dependence of core estimation. Diffusion-weighted imaging (DWI) with apparent diffusion coefficient (ADC) mapping, acquired 15 min later and segmented using an ADC threshold of <600 × 10^−6^ mm^2^/s, is shown as an imaging reference for the spatial extent of infarct core. The figure is based on original anonymized clinical imaging and does not derive from previously published material or external datasets. It is intended for educational purposes, illustrating how different processing strategies may influence perfusion map appearance and lesion-volume estimation, rather than as a quantitative validation or performance comparison of specific software implementations.

Algorithmic and technical factors may further affect map appearance. Acquisition parameters, reconstruction methods, image noise, postprocessing choices, and the deconvolution model itself can influence not only calculated lesion volumes but also the apparent spatial distribution of core and hypoperfused tissue [4,6,8]. Figure 5 illustrates how different algorithms applied to the same CTP study can generate divergent core and penumbra maps. This does not make automated perfusion maps unreliable, but reinforces the need to assess their outputs together with map quality and visual plausibility, rather than as isolated numerical results [32,33].

The role of CTP is therefore not uniform across reperfusion scenarios. In the conventional 4.5-h window, current guidelines emphasize rapid intravenous thrombolysis and recommend avoiding delays related to additional multimodal imaging, including this technique. Beyond 4.5 h, automated perfusion imaging may support selection for intravenous thrombolysis in patients with salvageable ischemic penumbra, including wake-up stroke within 9 h from the midpoint of sleep and patients 4.5–9 h from last known well. In suspected large vessel occlusion (LVO), rapid brain and vascular imaging remains central to endovascular thrombectomy selection. Adjunctive CTP or advanced magnetic resonance imaging may be useful between 6 and 24 h, if immediately available [3]. Accordingly, perfusion maps should be interpreted as tools that support clinical decisions within the broader imaging and clinical context, rather than as isolated arbiters of tissue viability [34,35]. This is particularly relevant for proprietary algorithms with closed source code, whose internal assumptions may not be fully transparent to clinicians but may still influence treatment eligibility based on thresholds.

Threshold interpretation is also time-dependent. In very early imaging, including the so-called golden hour, rCBF thresholds may overestimate irreversible injury, contributing to the ghost core phenomenon [36,37,38,39]. This further supports the need for cautious, context-aware interpretation of automated outputs. This should not be interpreted as disappearance of established infarct core, but rather as misclassification of severely hypoperfused yet still viable tissue by a threshold that depends on timing. In this setting, more restrictive CBF thresholds may better approximate irreversible injury, whereas the widely used rCBF < 30% threshold was largely validated in later or extended-window settings [38,40]. Conversely, with poor collateral flow or delayed imaging, the distinction between viable hypoperfused tissue and irreversible injury may progressively narrow [36,39].

Recent comparative and multicenter studies support this interpretation. Studies comparing different algorithms or software tools, including Bayesian, cSVD, and oSVD processing, as well as platforms such as RAPID, Olea Sphere, Viz.ai, Brainomix e-CTP, MIStar, and syngo.via, have shown that agreement varies according to the parameter, threshold, reference standard, and clinical decision rule [4,41,42,43,44,45,46].

In selected scenarios, treatment classifications may be broadly similar; however, relevant differences have been reported in estimated ischemic core, hypoperfused tissue, mismatch volume, spatial agreement, or thrombectomy eligibility [41,42,43,44,45,46]. These observations are consistent with previous acute stroke imaging roadmap recommendations and support standardization of ischemic region estimation across centers and platforms [15,47].

Table 3 summarizes selected CT perfusion software platforms and their reported or publicly described algorithmic approaches. The focus is limited to the deconvolution or perfusion-estimation model, rather than to the full proprietary post-processing pipeline, which may include undocumented, vendor-specific, and version-dependent steps. Because public information is heterogeneous across vendors, the table should be interpreted as an *educational overview* supported by source attribution, not as a definitive technical, regulatory, or version-specific specification. For this reason, software-specific statements were supported, whenever available, by three levels of evidence: regulatory documentation, peer-reviewed comparative or validation studies, and vendor technical material, white papers, product pages, or instructions for use. Regulatory documents were used primarily to verify public device identity, intended use, cleared functionality, and available outputs, whereas algorithmic statements were derived from peer-reviewed comparative literature or vendor technical documentation when regulatory sources did not disclose the underlying method. This distinction is relevant when comparing outputs across different commercial platforms and when interpreting threshold-dependent lesion volumes.

## 10. Artificial Intelligence in Perfusion Map Estimation

### 10.1. AI and Classical Model-Based Automation

Artificial intelligence (AI) is increasingly used in acute stroke imaging, but it should be clearly distinguished from classical model-based automation. In current clinical CT perfusion post-processing, the maps used for treatment selection are still generated predominantly through predefined mathematical models, including deconvolution, temporal correction, regularization, and software-specific thresholds. These processes may be highly automated, but they are not necessarily AI-based, because their outputs are primarily determined by explicit physiological and mathematical assumptions rather than by representations derived from training datasets.

Within the broader field of AI, it is useful to distinguish machine learning from deep learning. *Machine learning* refers to methods that identify patterns in data, whereas *deep learning* is a subset of machine learning based on multilayer neural networks designed to extract progressively more complex features. Deep learning includes several architectures and hybrid strategies, such as convolutional neural networks (CNNs), transformer-based models, generative models, and physics-informed approaches.

*AI already integrated into commercial stroke workflows*. In commercial stroke imaging platforms, AI is currently used mainly for tasks adjacent to perfusion map generation. Machine learning methods, including decision-tree-based approaches, have been explored for automated assessment of early ischemic changes on noncontrast CT using the Alberta Stroke Program Early CT Score (ASPECTS), whereas deep learning approaches, particularly CNNs, are increasingly applied to LVO detection on CTA. Other applications include image quality control, triage alerts, and workflow prioritization. These tools may accelerate diagnosis and communication, but their presence within a stroke platform does not imply AI-based generation of perfusion maps. A full review of AI across stroke imaging is beyond the scope of this article; the following sections therefore provide only a concise, perfusion-focused overview of selected emerging methods.

*AI methods and hybrid models for perfusion estimation*. Beyond workflow applications, recent research has begun to explore these strategies in perfusion analysis itself. These include: (i) CNN-based approaches, which may support direct map generation from perfusion data; (ii) transformer models, which may analyze temporal relationships in contrast passage curves; (iii) generative models, which may synthesize new imaging representations; (iv) physics-informed models, which may constrain learning through physiological rules; and (v) end-to-end pipelines, which may predict clinically relevant outputs while bypassing intermediate parametric maps. These approaches remain primarily research-oriented and are being investigated as alternatives or complements to classical deconvolution, rather than as standardized clinical replacements.

### 10.2. Neural Network Derived and Physics-Informed Perfusion Maps

Neural networks and hybrid learning strategies are being investigated to estimate perfusion maps or parameters directly from dynamic data, reducing dependence on voxel-wise deconvolution and model-based inversion.

*Neural network-derived maps*. CNN-based, model-free approaches have been proposed as alternatives to conventional deconvolution for perfusion map generation [76]. The model is trained to learn the relationship between dynamic input data and the corresponding parametric outputs, without explicitly estimating the residue function voxel by voxel. CNN-derived maps have shown strong agreement with conventional deconvolution outputs, suggesting that neural networks may approximate this process directly from the data. Potential advantages include greater use of spatial context and improved robustness in noisy or motion-prone datasets. However, when trained on software-derived outputs, these models may inherit the assumptions and errors of the reference algorithm.

*Physics-informed learning*. Hybrid methods constrain deep learning with physiological or mathematical priors rather than relying solely on data-driven estimation. A spatio-temporal perfusion physics-informed neural network, SPPINN, has been proposed to learn implicit representations of contrast attenuation and estimate continuous perfusion parameter maps [77]. In this model, training is guided by the known relationship between arterial input, tissue curves, and perfusion parameters. This preserves a link with the physical basis of perfusion imaging while improving robustness to noise. However, the extent to which such physics-informed constraints improve reproducibility across scanners, acquisition protocols, and patient populations remains to be established [78].

*Transformer-based parameter estimation*. Transformer models are neural networks designed to model relationships within sequences by weighting the relative importance of different time points or features. Applied to perfusion analysis, they can analyze contrast passage curves, where bolus arrival, temporal dependencies, and tissue response are central. CTPerformer Net, for example, incorporates physical consistency, smoothness, and physical model priors into the loss function [79]. Another transformer-based approach used a single arterial input curve and voxel wise concentration time curves to estimate the local arterial input function, the flow scaled residue function, CBF, and bolus arrival delay [80]. These models may reduce dependence on a single arterial reference curve and improve handling of delay, dispersion, and noisy residue functions.

### 10.3. End to End and Generative Approaches

Some AI approaches bypass conventional tracer kinetic analysis altogether. Rather than generating CBF, CBV, MTT, Tmax, or Delay maps, they attempt to predict clinically relevant endpoints directly from native perfusion data.

*End to End Prediction*. End to end learning is not a specific neural network architecture, but a strategy in which the model maps input data directly to the desired output, bypassing intermediate parametric maps. In this context, end to end CNN models have been proposed to classify ischemic core volume from raw slice reduced perfusion data [81], whereas deconvolution free models have been used to predict final infarct volume from native perfusion images and clinical metadata [82]. These approaches may capture tissue fate and treatment effects more directly, but they are less transparent than conventional CBF, CBV, Tmax, Delay, and mismatch maps.

*Generative Synthetic Perfusion Mapping*. Generative models are based on the assumption that a network can learn the statistical relationship between an input imaging domain and a target imaging representation, and then synthesize a plausible output in the target domain. In this context, physiology informed generative multitask networks have been investigated to generate multiple synthetic contrast free perfusion maps from noncontrast CT [83]. This strategy derives perfusion-like information from structural CT rather than from dynamic contrast passage. Such methods may be useful when full perfusion imaging is unavailable or operationally difficult, but synthetic outputs remain dependent on the data, target maps, and procedures used for training.

### 10.4. Clinical Implications

AI methods and hybrid approaches should currently be regarded as complements to classical deconvolution, not as replacements. They may improve robustness to noise, reduce dependence on arterial input selection, incorporate spatial and temporal context, and support local hemodynamic estimation. However, conventional algorithms remain essential, because they still underlie most perfusion maps used for treatment selection.

## 11. Educational Pearls and Pitfalls for CT Perfusion Interpretation

Table 4 provides a practical checklist for perfusion map interpretation, focusing on acquisition quality, processing model, timing metrics, threshold selection, and clinical imaging integration. It is intended to support a structured reading of automated outputs before they are translated into treatment-relevant estimates of core, hypoperfusion, and mismatch. This approach emphasizes that perfusion interpretation should begin with verification of data quality and methodological context, rather than with isolated volumetric thresholds. It also reinforces the need to interpret automated maps alongside noncontrast CT, CTA, vascular anatomy, and the clinical time window.

## 12. Limitations

This review has some limitations. *First*, it is intended as a clinically oriented technical review rather than a systematic review, and no formal meta-analysis was performed. For the same reason, advanced mathematical formulations and explicit matrix representations were intentionally limited to preserve readability for a clinical audience. *Second*, several CT perfusion software implementations are proprietary, may vary across software releases, and are incompletely described in publicly available sources, limiting the level of technical detail that can be reported. *Third*, the illustrative imaging example is not intended to provide independent validation, volumetric comparison, Dice-based agreement analysis, runtime assessment, or vendor-specific benchmarking of any algorithm. Its purpose is limited to visually demonstrating how different processing strategies may influence perfusion map interpretation in a single clinical scenario.

*Finally*, although this review emphasizes algorithmic and threshold variability, treatment decisions in acute ischemic stroke remain dependent on clinical presentation, imaging quality, vascular findings, local workflow, and guideline-based criteria.

## 13. Conclusions

CTP provides a valuable window into cerebral hemodynamics, but its clinical value depends on contextual interpretation rather than isolated map reading. Findings should be integrated with clinical presentation, noncontrast CT, CTA, occlusion site, collateral status, acquisition quality, and imaging-to-treatment context [34,35,47]. Algorithms should support, not replace, clinical judgment. Future work should prioritize transparent reporting of software versions, assumptions, thresholds, and cross-platform validation.

## Figures and Tables

**Table 1:** Main perfusion maps: physiological meaning, origin, and algorithmic dependencies.

Map	Physiological Meaning	Derived From	Deconvolution	Algorithm/Model
**CBF**	Cerebral Blood Flow (mL/100 g/min)	Maximum height of R(t)	Yes	sSVD, bcSVD, oSVD, ddSVD, Bayesian
**CBV**	Cerebral Blood Volume (mL/100 g)	Area under the tissue concentration curve normalized to the arterial input	No	Direct from concentration–time curve
**MTT**	Average time required for blood to traverse the tissue microvasculature	Area under R(t), or indirectly CBV/CBF according to the central volume theorem	Optional; deconvolution-based estimation preferred	sSVD, bcSVD, oSVD, ddSVD, Bayesian
**Tmax**	Composite timing metric of the tissue residue response	Time to maximum of R(t)	Yes	SVD-based methods; model-dependent implementations
**Delay**	Arterial–tissue arrival delay	Temporal shift between AIF and tissue response	Yes, when explicitly estimated within the model	ddSVD, Bayesian/model-based approaches
**TTP (classic)**	Timing of maximal tissue enhancement	Peak of the raw tissue concentration curve	No	Direct, no model correction; highly delay-sensitive
**TTP (model-based)**	Timing of maximal tissue enhancement	Peak of a regularized or model-estimated tissue curve	Yes/model-based	Bayesian/model-based implementation

**Table 2:** Main deconvolution algorithms: delay sensitivity and key technical features.

Algorithm	Delay Sensitivity	Key Technical Features
**sSVD** Standard SVD	Sensitive	*Linear deconvolution* with fixed temporal boundaries, treating the sampled time curve as having a defined beginning and end. It assumes that arterial input precedes the tissue signal. It is fast and simple, but highly delay-sensitive and prone to perfusion underestimation.
**bcSVD** Block-circulant SVD	Reduced compared with sSVD	A *circular formulation* mathematically links the end of the time curve to its beginning. The convolution matrix is organized into coupled sub-matrices (“*blocks*”). This improves numerical stability and reduces sensitivity to bolus delay, at the cost of an implicit, non-physiological periodicity assumption.
**oSVD** Oscillation-index SVD	Reduced compared with sSVD	Extension of bcSVD with oscillation-index *regularization*. Iteratively suppresses spurious oscillations in R(t), yielding smoother and more stable maps; computationally more demanding, without explicit dispersion correction.
**ddSVD** Delay- and dispersion-corrected SVD	Corrected for delay and dispersion	Explicit correction for both *delay*, via cross-correlation realignment, and *dispersion*, through vascular dispersion kernel modeling. Provides a more physiologically informed estimate of R(t), especially in collateral flow, but with increased complexity.
**Bayesian**	Model-based; reduced sensitivity to delay and dispersion effects	*Probabilistic estimation* of R(t) constrained by physiological priors. Selects the most likely solution from the observed data and prior assumptions, improving robustness to noise, delay, and truncation, with increased model complexity.

**Table 3:** Educational summary of reported algorithmic approaches in selected software.

Vendor/Platform	Reported Approach	Notes and Supplementary References
**RAPID** (iSchemaView, Menlo Park, CA, USA)	Fourier transform-based deconvolution; also described as delay-insensitive, with one practical workflow description referring to *circular deconvolution*	Widely adopted automated CTP platform. Full proprietary implementation and explicit dispersion-correction strategy are not publicly disclosed [48,49,50,51]
**Olea Sphere** (Olea Medical, France)	sSVD, cSVD, oSVD, Bayesian	Supports multiple perfusion post-processing models, including SVD-based variants and Bayesian probabilistic estimation; cSVD denotes block-circulant SVD [4,52,53]
**MIStar** (Apollo Medical Imaging, Australia)	Delay- and dispersion-corrected deconvolution, commonly described as ddSVD	ddSVD corrects for both bolus delay and dispersion effects, although detailed implementation remains proprietary. It also provides Delay Time, DT, as an algorithm-specific timing metric [54,55,56]
**Vitrea** (Canon Medical, MN, USA)	sSVD, SVD+ (delay-corrected), Bayesian	Reported to include standard SVD, SVD+ as a delay-corrected SVD variant, and Bayesian estimation; detailed implementation remains proprietary [57,58,59]
**Brainomix e-CTP** (Oxford, UK)	Block-circulant deconvolution	Block-circulant deconvolution with reduced delay sensitivity; thresholds are user-configurable [60,61,62]
**GE Healthcare** (GE Healthcare, Chicago, IL, USA)	Delay-corrected deconvolution	Delay-corrected approach. Detailed implementation remains proprietary and may vary across software releases [63,64,65]
**Philips IntelliSpace Portal** (Philips Healthcare, Best, The Netherlands)	ATS (Arrival-Time-Sensitive SVD), ATI (Arrival-Time-Insensitive SVD)	Based on analytical and SVD-derived deconvolution concepts; ATI represents an arrival-time-insensitive implementation [66,67,68]
**Siemens syngo.via** (Siemens Healthineers, Erlangen, Germany)	Deconvolution (DC), Maximum Slope (MS)	DC represents a deconvolution-based method with reduced delay sensitivity; MS relies on peak-slope analysis. Detailed implementation remains proprietary and may vary across software releases [69,70,71]
**VEObrain** (Germany)	Regularized deconvolution (Tikhonov)	Uses Tikhonov regularization to stabilize deconvolution and reduce noise-related oscillations; explicit delay or dispersion correction is not clearly documented [72,73]
**Viz.ai** (San Francisco, USA)	Not publicly disclosed	AI-enabled workflow platform; CTP deconvolution strategy and thresholds not fully publicly disclosed [48,74,75]

References labeled [4,48,49,50,51,52,53,54,55,56,57,58,59,60,61,62,63,64,65,66,67,68,69,70,71,72,73,74,75] refer to Supplementary File S1: Source attribution for software-specific CT perfusion algorithmic descriptions. Absence of a disclosed algorithmic detail in public documentation should not be interpreted as absence of that processing step in the proprietary software pipeline.

**Table 4:** Practical checklist for clinical interpretation of perfusion maps.

Step	Practical Point
1	*Check acquisition quality first*. Motion, truncation, poor bolus timing, low signal-to-noise ratio, or inadequate vascular curve selection can compromise derived maps.
2	*Identify the processing model*. Interpretation should consider whether the software uses SVD-based, delay-corrected, delay- and dispersion-corrected, Bayesian, or other approaches.
3	*Separate direct from deconvolution-derived parameters*. CBV and classic TTP are linked to the tissue curve; CBF, MTT, and Tmax depend on the residue response; Delay may be estimated separately or incorporated within the model.
4	*Do not transfer thresholds across platforms*. rCBF, Tmax, Delay/DT, and TTP thresholds are operational cut-offs, not interchangeable physiological constants.
5	*Interpret core as an estimate, not a certainty*. Ischemic core volume depends on the algorithm, threshold, imaging time, and reperfusion status.
6	*Consider the treatment window*. Early rCBF thresholds may overestimate core; with delayed imaging, the distinction between viable and infarcted tissue may narrow.
7	*Assess delay and dispersion*. Delayed arrival and bolus dispersion can distort perfusion estimates, especially in proximal occlusion or collateral-dependent flow.
8	*Compare maps with CT angiography (CTA) and non-contrast CT*. Occlusion site, collaterals, early ischemic changes, mass effect, and vascular anatomy should guide interpretation.
9	*Look for mimics and confounders*. Seizure, migraine, chronic stenosis, reperfusion, vascular variants, tumor-related hyperperfusion, and technical artifacts may alter perfusion maps.
10	*Use automation as decision support*. Automated maps can accelerate interpretation, but treatment decisions should integrate clinical severity, timing, vascular findings, image quality, and workflow.

## Data Availability

No new data were created or analyzed in this study. Data sharing is not applicable to this article.

## Supplementary Materials

The following supporting information can be downloaded at https://www.mdpi.com/article/10.3390/diagnostics16121831/s1, File S1: Source attribution for software-specific CT perfusion algorithmic descriptions.

## Abbreviations

The following abbreviations are used in this manuscript:

ADC	Apparent diffusion coefficient
AIF	Arterial input function
AI	Artificial intelligence
ASPECTS	Alberta Stroke Program Early CT Score
ATI	Arrival-time-insensitive
ATS	Arrival-time-sensitive
bcSVD	Block-circulant singular value decomposition
CBF	Cerebral blood flow
CBV	Cerebral blood volume
cSVD	Circulant singular value decomposition
CNN	Convolutional neural network
CT	Computed tomography
CTA	Computed tomography angiography
CTP	Computed tomography perfusion
C(t)	Tissue concentration curve
DC	Deconvolution
ddSVD	Delay- and dispersion-corrected singular value decomposition
DT	Delay Time
DWI	Diffusion-weighted imaging
HU	Hounsfield units
LVO	Large vessel occlusion
MS	Maximum slope
MTT	Mean transit time
oSVD	Oscillation-index regularized singular value decomposition
rCBF	Relative cerebral blood flow
R(t)	Residue function
SVD	Singular value decomposition
SPPINN	Spatio-temporal perfusion physics-informed neural network
sSVD	Standard singular value decomposition
TCC	Tissue time-concentration curve
TDC	Time-density curve
Tmax	Time to maximum
TTP	Time to peak
VOF	Venous output function
