# Supplementary material for: Understanding CT Perfusion in Acute Ischemic Stroke: How Algorithms Shape Perfusion Maps
Source: Diagnostics (Basel). 2026 Jun 12;16(12):1831. doi: 10.3390/diagnostics16121831 (PMC13297754; doi:10.3390/diagnostics16121831)
Supplement: Supplementary file 1 [file diagnostics-16-01831-s001.zip › diagnostics-4325852-supplementary.pdf]

Supplementary File S1.
Source attribution for software-specific CT perfusion algorithmic descriptions

This supplementary file provides source attribution for the software-specific algorithmic descriptions summarized in Table 3. References are grouped by software platform and numbered progressively as [4,48–75]. For each platform, whenever available, three levels of evidence were prioritized: regulatory documentation, peer-reviewed comparative or validation studies, and vendor technical material, white papers, product pages, practical workflow descriptions, or instructions for use. Regulatory documents were used primarily to verify public device identity, intended use, cleared functionality, and available outputs. When regulatory documents did not disclose the underlying algorithm, algorithmic statements were derived from peer-reviewed comparative literature or vendor technical documentation, where available. Because public technical information is heterogeneous across vendors, this source list should be interpreted as structured attribution for an educational summary rather than as a complete disclosure of proprietary software implementation.

RAPID / RapidAI / iSchemaView

Comment: Sources support the description of RAPID as Fourier transform–based deconvolution, also described as delay-insensitive in comparative literature, with one practical workflow description referring to circular deconvolution. FDA documentation is used to verify the public regulatory description of the software and available functionality, but does not provide full proprietary algorithmic disclosure.

[48] Bushnaq S, Hassan AE, Delora A, Kerro A, Datta A, Ezzeldin R, et al. A Comparison of CT Perfusion Output of RapidAI and Viz.ai Software in the Evaluation of Acute Ischemic Stroke. *AJNR Am J Neuroradiol.* **2024**;45:863–870.
[49] Austein F, Riedel C, Kerby T, Meyne J, Binder A, Lindner T, et al. Comparison of Perfusion CT Software to Predict the Final Infarct Volume After Thrombectomy. *Stroke. 2016*;47:2311–2317.
[50] Practical Neurology. RAPID Automated CT Perfusion in Clinical Practice. *Practical Neurology*. **2019**. Available from: https://practicalneurology.com/diseases-diagnoses/imaging-testing/rapid-automated-ct-perfusion-in-clinical-practice/31600/. Accessed June 2, 2026.
[51] U.S. Food and Drug Administration. iSchemaView RAPID. 510(k) Premarket Notification K182130. Silver Spring, MD: U.S. Food and Drug Administration; 2018. Available from: https://www.accessdata.fda.gov/cdrh_docs/pdf18/K182130.pdf. Accessed June 2, 2026.

Olea Sphere / Olea Medical

Comment: Sources support the presence of Bayesian probabilistic perfusion estimation and SVD-based approaches within Olea Sphere. The peer-reviewed CT perfusion-specific source documents the comparison of Bayesian, cSVD, and oSVD algorithms in Olea Sphere for infarct-volume prediction in patients with acute ischemic stroke.

[52] Olea Medical. The Bayesian Saga. Olea Medical; 2016. Available from: https://www.olea-medical.com/wp-content/uploads/2016/03/01-Olea-Imagein-The-Bayesian-Saga.pdf. Accessed June 2, 2026.
[53] U.S. Food and Drug Administration. Olea Medical CT Perfusion V1.0. 510(k) Premarket Notification K223091. Silver Spring, MD: U.S. Food and Drug Administration; 2023. Available from: https://www.accessdata.fda.gov/cdrh_docs/pdf22/K223091.pdf. Accessed June 2, 2026.

[4] Yao Y, Gu S, Liu J, Li J, Wu J, Luo T, Li Y, Ge B, Wang J. Comparison of Three Algorithms for Predicting Infarct Volume in Patients with Acute Ischemic Stroke by CT Perfusion Software: Bayesian, cSVD, and oSVD. *Diagnostics*. **2023**;13:1810.

MIStar / AutoMIStar / Apollo Medical Imaging Technology

Comment: Sources support MIStar/AutoMIStar as a delay- and dispersion-corrected deconvolution approach, commonly described as ddSVD. FDA documentation specifically supports Delay Time as an algorithm-specific timing metric derived from an improved delay- and dispersion-corrected deconvolution method.

[54] Gunasekera L, Churilov L, Mitchell P, Dowling RJ, Kleinig TJ, Yan B. Automated estimation of ischemic core prior to thrombectomy: comparison of two current algorithms. *Neuroradiology*. **2021**;63:1645–1649.
[55] U.S. Food and Drug Administration. AutoMIStar. 510(k) Premarket Notification K192912. Silver Spring, MD: U.S. Food and Drug Administration; 2019. Available from: https://www.accessdata.fda.gov/cdrh_docs/pdf19/K192912.pdf. Accessed June 2, 2026.
[56] Apollo Medical Imaging Technology Pty Ltd. AutoMIStar CTP Stroke. Apollo Medical Imaging Technology Pty Ltd. Available from: https://www.apollomit.com/automistar.htm. Accessed June 2, 2026.

Vitrea / Canon Medical Systems

Comment: Sources support the availability of standard SVD, SVD+ and Bayesian CT perfusion post-processing within Vitrea/Canon material. FDA documentation specifically supports the addition of the Bayesian algorithm and the availability of Bayesian and SVD+ options.

[57] Canon Medical Systems Corporation. Bayesian CT Perfusion Imaging in Ischemic Stroke. Canon Medical Systems Corporation; 2021. Available from: https://academy.eu.medical.canon/wp-content/uploads/2025/06/MOIHI0003EAC_high-Bayesian-CT-Perfusion-Imaging-in-Ischemic-Stroke.pdf. Accessed June 2, 2026.
[58] Canon Medical Systems Corporation. CT Brain Perfusion. Canon Medical Systems Corporation. Available from: https://global.medical.canon/products/healthcare_it/clinical_application/ct_brain_perfusion. Accessed June 2, 2026.
[59] U.S. Food and Drug Administration. Vitrea CT Brain Perfusion. 510(k) Premarket Notification K181247. Silver Spring, MD: U.S. Food and Drug Administration; 2018. Available from: https://www.accessdata.fda.gov/cdrh_docs/pdf18/K181247.pdf. Accessed June 2, 2026.

Brainomix 360 e-CTP / Brainomix e-Stroke

Comment: Sources support Brainomix 360 e-CTP as an automated CT perfusion module. FDA documentation supports block-circulant deconvolution, while peer-reviewed real-world data support comparison of e-CTP outputs with syngo.via. Vendor material supports automated CTP estimation of core, penumbra and mismatch metrics with configurable thresholds.

[60] U.S. Food and Drug Administration. Brainomix 360 e-CTP. 510(k) Premarket Notification K223555. Silver Spring, MD: U.S. Food and Drug Administration; 2023. Available from: https://www.accessdata.fda.gov/cdrh_docs/pdf22/K223555.pdf. Accessed June 2, 2026.
[61] Mallon D, Fallon M, Blana E, McNamara C, Menon A, Ip CL, et al. Real-world evaluation of Brainomix e-Stroke software. *Stroke Vasc Neurol*. **2024**;9:e002859.

[62] Brainomix. e-CTP: AI Decision Support Software for CT Perfusion Imaging. Brainomix. Available from: https://www.brainomix.com/stroke/e-ctp/. Accessed June 2, 2026.

GE CT Perfusion 4D / CT Perfusion 4D Neuro

Comment: Sources support GE CT Perfusion 4D as CT perfusion post-processing software. FDA documentation supports the regulatory identification of FastStroke/CT Perfusion 4D. Peer-reviewed literature documents use of CT Perfusion 4D on GE Advantage Workstation and describes it as deconvolution-based. Vendor technical material supports the delay-corrected deconvolution wording used in Table 3.

[63] U.S. Food and Drug Administration. FastStroke, CT Perfusion 4D. 510(k) Premarket Notification K193289. Silver Spring, MD: U.S. Food and Drug Administration; 2020. Available from: https://www.accessdata.fda.gov/scripts/cdrh/cfdocs/cfpmn/pmn.cfm?ID=K193289. Accessed June 2, 2026.
[64] GE HealthCare. CT Perfusion 4D Neuro. GE HealthCare product data sheet. Available from: https://www.gehealthcare.com/content/dam/gehc/sitecore-migrated-assets/widen/2018/01/25/0204/gehealthcarecom/migrated/2018/02/19/0837/tion-product-spec-sheets-ct-perfusion-4d-neuro-gehc-datasheet_aw-ct-perfusion-4d-neuro_pdf. Accessed June 2, 2026.
[65] Lei L, Zhou Y, Guo X, Wang L, Zhao X, Wang H, Ma J, Yue S. The value of a deep learning image reconstruction algorithm in whole-brain computed tomography perfusion in patients with acute ischemic stroke. *Quant Imaging Med Surg*. **2023**;13:8173–8189.

Philips IntelliSpace Portal / CT Brain Perfusion

Comment: Sources support Philips IntelliSpace Portal / CT Brain Perfusion as a platform in which arrival-time-sensitive and arrival-time-insensitive approaches are described or evaluated. FDA documentation is platform-level and does not provide a full CT perfusion algorithmic disclosure. Philips instructions for use describe the Brain Perfusion application, its calculated parameters, and the distinction between time-arrival-sensitive and time-arrival-insensitive methods.

[66] U.S. Food and Drug Administration. IntelliSpace Portal Platform. 510(k) Premarket Notification K162025. Silver Spring, MD: U.S. Food and Drug Administration; 2016. Available from: https://www.accessdata.fda.gov/cdrh_docs/pdf16/K162025.pdf. Accessed June 2, 2026.
[67] Pennig L, Thiele F, Goertz L, et al. Comparison of Accuracy of Arrival-Time-Insensitive and Arrival-Time-Sensitive CTP Algorithms for Prediction of Infarct Tissue Volumes. *Sci Rep*. **2020**;10:9252.
[68] Philips. IntelliSpace Portal CT Analysis. Instructions for Use. Document 300006524241_A. Philips; 2021. Available from: Philips Document Library, https://www.documents.philips.com/assets/Instruction%20for%20Use/20210412/d652a0ac1db946e8b236ad080070403c.pdf/ Accessed June 2, 2026.

Siemens syngo.via / syngo.CT Neuro Perfusion

Comment: Sources support syngo.CT Neuro Perfusion as a CT perfusion software package with deconvolution and Maximum Slope approaches described in Siemens technical material. Peer-reviewed literature supports the use of syngo.via CT Neuro Perfusion and documents software-specific core-estimation approaches.

[69] U.S. Food and Drug Administration. syngo.CT Neuro Perfusion. 510(k) Premarket Notification K202213. Silver Spring, MD: U.S. Food and Drug Administration; 2020. Available from: https://www.accessdata.fda.gov/cdrh_docs/pdf20/K202213.pdf. Accessed June 2, 2026.

[70] Hoving JW, Koopman MS, Tolhuisen ML, van Voorst H, Brehm M, Berkhemer OA, et al. Accuracy of CT perfusion ischemic core volume and location estimation: a comparison between four ischemic core estimation approaches using syngo.via. *PLoS One*. **2022**;17:e0272276.
[71] Siemens Healthineers. CT Neuro Perfusion in Ischemic Stroke Management. Siemens Healthineers. Available from: https://academy.siemens-healthineers.com/_/en-us/ct-neuro-perfusion-in-ischemic-stroke-management/. Accessed June 2, 2026.

VEObrain / VEOcore

Comment: Sources support VEOcore as a Tikhonov-regularized deconvolution approach with automated motion correction, denoising and quality control. Vendor documentation describes VEOcore as CE-labelled, but no publicly accessible CE/MDR certificate with class, certificate number and notified body was identified.

[72] Rau A, Reisert M, Stein T, Mueller-Peltzer K, Rau S, Bamberg F, Taschner CA, Urbach H, Kellner E. Impact of temporal resolution on perfusion metrics, therapy decision, and radiation dose reduction in brain CT perfusion in patients with suspected stroke. *Neuroradiology*. **2024**;66:749–759.
[73] VEObrain GmbH. VEOcore. VEObrain GmbH. Available from: https://www.veobrain.com/?lang=en&page=veocore. Accessed June 2, 2026.

Viz.ai / Viz CTP

Comment: Sources support Viz CTP as an automated CTP-processing and communication software package. Public FDA and vendor documentation describe functionality and outputs but do not disclose the specific perfusion deconvolution implementation. Comparative literature [A1] explicitly reports that implementation details were not identified in the authors' literature search.

[74] U.S. Food and Drug Administration. Viz CTP. 510(k) Premarket Notification K180161. Silver Spring, MD: U.S. Food and Drug Administration; 2018. Available from: https://www.accessdata.fda.gov/cdrh_docs/pdf18/K180161.pdf. Accessed June 2, 2026.
[75] Viz.ai. Viz CTP. Viz.ai. Available from: https://www.viz.ai/ct-perfusion. Accessed June 2, 2026.
